# Some Hospitals and Municipal Institutions

**Published:** 1914-06-27

**Authors:** Johnstone Wallace


### THE BRITISH HOSPITALS ASSOCIATION.

### SOME HOSPITALS AND MUNICIPAL INSTITUTIONS.

### By The Lord Mayor of Newcastle, Councillor JOHNSTONE WALLACE, J.P.

May I, on behalf of the Conference, express to you, Sir George Hare Philipson, our thanks for the kindly words of welcome which you have been good enough to give us, and also to the house committee of this infirmary for having been so good as to house us so comfortably and so conveniently as they have done. I may say to you, gentlemen, that Sir George Hare Philipson has been for fifty-six years associated officially with this great institution, and that he is one of our great local assets. I express cordially our thanks to him for those words of welcome. It is an especial pleasure to extend cordial greetings to the representatives of an Association like the British Hospitals Association, to whose deliberations are so much due improved methods of hospital management, greater efficiency in their administration, and the wider diffusion of knowledge in relation to medical relief generally. And, surely, no more suitable place could be found for the holding of this, the fifth, annual Conference of the Association than the place where we are now met together. The foundation-stone of this magnificent institution was laid, in the year 1900, by our late beloved King Edward VII., when, as Prince of Wales, he visited this city as the representative of Queen Victoria, and six years later the buildings were opened by his late Majesty King Edward, the then reigning monarch. The keen interest which our Royal Family have ever evinced in hospital work was therefore particularly marked with regard to this institution. And the circumstances prior to the foundation-stone laying ceremony, and subsequently thereto, justified the Royal recognition, for this Queen Victoria Infirmary has been throughout a monument to local philanthropy and to the generous support of the people generally—and particularly, I might add, of the working classes themselves. This is not the time to dwell in detail upon the history of the change of site of the old infirmary near the Central Railway station and cattle docks to this more salubrious situation, but it will doubtless be remembered by many here to-day how the present building had its inception first by a Diamond Jubilee scheme, initiated by the then Mayor, Mr. (now Sir) Riley Lord, to raise a sum of £60,000, representing £1,000 for each year of Queen Victoria's reign, which sum of £60,000 was subsequently raised to £100,000 by a strenuous campaign in the course of which the working classes themselves contributed the handsome sum of £20,000. Then the munificent gift of £100,000 by the late Mr. John Hall, conditionally on the change of site to the Castle Leazes or Town Moor, which change was facilitated in every possible way by the City Council and the Stewards' Committee of the Freemen; and finally the princely gift of a further £100,000 by Mr. W. A. Watson Armstrong (now Lord Armstrong) and his wife, without restriction with regard to its application for either building or endowment purposes. As a result, we have here an up-to-date infirmary, which is probably second to none in the whole country; and I venture to say that where we have such widespread consideration for the suffering poor as has been thus evinced in connection with its establishment, there is still much that is good and Christ-like in the hearts of the people. The ladies and gentlemen attending this Conference will find that Newcastle is wonderfully well provided with institutions for the relief of the suffering members of the community; but one really needs to fill a position such as that of Lord Mayor to realise the self-sacrifice of such a large number of our fellow-citizens, both professional and lay, in the interests of our less fortunate brothers and sisters. And before proceeding further may I be permitted to mention a little matter which has been often on my mind? The immense advantage gained by the introduction of trained nurses was generally admitted. The nurses who are daily moving about in our midst are a constant reminder to us of the noble profession which they have adopted. There is something so quiet and unassuming about their uniform that appeals to our sympathies and commands our respect, and, if not out of place, I would suggest that steps should be taken by your Association to ensure that as far as ever practicable that uniform shall never be brought into disrepute by being allowed to be worn by any persons other than those duly entitled to wear it. The work of voluntary hospitals is to be dealt with by the Sheriff and Dr. Hume, whose associations with these matters for many years past qualify them to speak with authority on the subject.

### Municipal Hospitals of Newcastle.

But, whilst voluntary hospitals will be chiefly in your mind during this Conference, I may perhaps be permitted to make a brief reference to hospitals in the management of the Corporation which by the kindness of my colleagues I have the honour to represent. As trustees of the hospital of St. Mary Magdalene, the Corporation, through their Schools and Charities Committee, control what is known as the St. Mary Magdalene Home for Incurables. I believe this hospital is the only one of its kind between Harrogate and Edinburgh. The hospital itself, originally established in the early part of the twelfth century, was a priory or hospital for a master, brethren, and sisters, to receive persons afflicted with leprosy, then a great scourge in this country. That priory occupied a site in Barras Bridge, where St. Thomas's Church is now to be seen. St. Thomas's Church itself, which is the chapel attached to the hospital, I do not need to deal with, and for the same reason I do not propose to refer in detail to the almshouses or to other matters connected with the Magdalene Hospital Charity. What will be of particular interest to the members of the Conference is the Home for Incurables, as it now stands, at Spital Tongues, within half a mile of this institution. At that "home"—a term which we like to emphasise in referring to the place—we receive seventy-two adult patients (thirty-six of either sex) in the main building, which was re-erected in 1884; and in the new wing, called the "Richardson wing," after the late esteemed chairman of the committee, Alderman Thomas Richardson, accommodation is provided for twenty-eight children, although fewer than half that number of children are yet in residence. Additional accommodation has recently been arranged for the female patients. The patients, whether adult or juvenile, must be hopelessly disqualified, by disease or deformity, for the duties of life; and I unhesitatingly say that there is no more soul-satisfying aspect of municipal life than the administration and visitation of this hospital, occupied as it now is by paralytics and sufferers from rheumatoid arthritis and other incapacitating diseases, notwithstanding which the patients will tell you that they are happy and comfortable. Then we have at Walkergate our City Hospital for Infectious Diseases, built in 1888, and at that time and for some years afterwards considered to be one of the most up-to-date fever hospitals in the kingdom. That building is under the immediate control of the Corporation Sanitary Committee, ably presided over by our friend, Alderman Sir H. W. Newton. The hospital was enlarged in 1908, contains 176 beds, and is now about to be further enlarged by the addition of sixty beds which will be especially allocated to cases of advanced tuberculosis. Further accommodation is also available for infectious diseases in temporary buildings, but in my opinion the sooner the structure is removed the better, for it is a blight on the Town Moor.

### The School Clinics.

The Corporation, through their Education Committee, ever mindful of the duties devolving upon them in common with other similar authorities, have also due regard to the health conditions of the children attending the Council schools. The eyesight, the teeth, and the general physical condition receive careful observation. And, in cases where necessitous circumstances justify it, the children are fed so that they may be able to reap due advantage from the lessons taught. The education authority employ three medical men in the school medical service. By the courtesy of Dr. Foggin, our education medical officer, I am able to state that during the past year (1913) the medical staff have carried out formal medical examinations of over 12,000 children in the elementary schools of this city. In addition to this the nurses in their visits to the various schools have examined, chiefly for cleanliness, over 20,000 children, and have issued notices to about 1,000 parents calling attention to defective conditions which they found. There are three clinics established—namely, one in the East End, one in the West End, and one in the central portion of the city, and at these the medical staff have examined and reported upon 3,730 cases. Whilst it has frequently been alleged that school clinics destroy parental responsibility, it is claimed by those actually associated with the work that the contrary is the case, and that the sense of parental responsibility is quickened through their being made aware of the danger of many so-called minor ailments of their offspring. In these ways the Corporation trust that, by looking ahead, they are not inconsiderably lessening the future burdens of the hospitals whose more efficient administration is of so much concern to you all. But whilst indulging in this reference to municipal management of hospitals and school clinics, and sincerely appreciating my privilege of association with the work, I must confess that I would be a poor advocate of State and municipal control of hospitals in general as against their voluntary management. It seems to me that in the case of voluntary hospitals we get a degree of soul-activity—genuine human sympathy—which would never be maintained under conditions of State management, which, in my opinion, would ultimately develop into a system of frigid bureaucracy. With many speakers to follow I do not propose to further dilate on hospital work from the municipal point of view, except to say that we rejoice that we have in our midst, as citizens, some of the most public-spirited medical men that the country can produce; that we are glad to see you here to confer with them and with the lay workers on matters of such moment; that we wish you the best of results in your investigations; and that, when the business part of your visit allows, you may find time to look around the city with a view to taking away with you happy recollections of your stay on Tyneside.

Dr. D. J. Mackintosh, M.B., LL.D., M.V.O., Chairman of the Council, tendered to the Lord Mayor their cordial thanks for his interesting and instructive address and for the hospitality which he was to extend to them during their visit to Newcastle. He added that they had no honours to offer to his lordship but one, and that was that he be President of the British Hospitals Association for the ensuing year. He hoped that the Lord Mayor would accept their humble offer, which was made with the best intentions, and that he would be their President and that they might have his judgment and advice during the year.

The Lord Mayor:

Dr. Mackintosh,—There is no need to thank me. I have considered it my duty to come here and say what was within my heart with regard to one or two matters that have for a long time interested me, and it has been a great pleasure to meet you all this morning. The duties of a Lord Mayor are many and varied, and the calls made upon him are numerous. Honours generally come thick upon one who has to act as Lord Mayor, but I always find that with those honours there are certain responsibilities. These make a man pause, because I do not know how far those responsibilities may extend, and I should not like anything to happen, or to undertake anything that I should feel I was not able to carry through. I accept with gratitude the position of President of your Association, and thank you for the honour which you have done me. The Lord Mayor then referred to the death, which had just taken place, of Alderman Richard Henry Holmes, of Newcastle, at the age of seventy-eight, who, he said, was the originator forty-five years ago of what was known nowadays as the Hospital Sunday Fund. Alderman Holmes toiled hard and long in furthering the interests of that fund, which was now so widespread throughout the world.

